# The Unfolded Protein Response as a Guardian of the Secretory Pathway

**DOI:** 10.3390/cells10112965

**Published:** 2021-10-31

**Authors:** Toni Radanović, Robert Ernst

**Affiliations:** 1 Medical Biochemistry and Molecular Biology, Medical Faculty, Saarland University, 66421 Homburg, Germany; Toni.Radanovic@uks.eu; 2 Preclinical Center for Molecular Signaling (PZMS), Medical Faculty, Saarland University, 66421 Homburg, Germany

**Keywords:** UPR, IRE1, PERK, ATF6, ER, lipid bilayer stress, proteotoxic stress, secretory pathway, hydrophobic mismatch, membrane thickness, membrane stiffness

## Abstract

The endoplasmic reticulum (ER) is the major site of membrane biogenesis in most eukaryotic cells. As the entry point to the secretory pathway, it handles more than 10,000 different secretory and membrane proteins. The insertion of proteins into the membrane, their folding, and ER exit are affected by the lipid composition of the ER membrane and its collective membrane stiffness. The ER is also a hotspot of lipid biosynthesis including sterols, glycerophospholipids, ceramides and neural storage lipids. The unfolded protein response (UPR) bears an evolutionary conserved, dual sensitivity to both protein-folding imbalances in the ER lumen and aberrant compositions of the ER membrane, referred to as lipid bilayer stress (LBS). Through transcriptional and non-transcriptional mechanisms, the UPR upregulates the protein folding capacity of the ER and balances the production of proteins and lipids to maintain a functional secretory pathway. In this review, we discuss how UPR transducers sense unfolded proteins and LBS with a particular focus on their role as guardians of the secretory pathway.

## 1. Introduction

The endoplasmic reticulum (ER) spans eukaryotic cells as a membrane-bound organelle with functionally and structurally distinct subdomains. Its membrane includes both the nuclear envelope (NE) separating the nucleoplasm from the cytoplasm, and the peripheral ER forming an elaborate network of tubules and cisternae [1]. The ER has important functions in cellular signaling, secretion, membrane protein biogenesis, and lipid metabolism. In most eukaryotic cells, the ER acts as a storage compartment for intracellular Ca^2+^, which can be released into the cytosol as an important second messenger in a wealth of signaling pathways [2]. A large portion of the ER surface contributes to membrane contact sites (MCSs), which provide a physical link to other organelles for exchanging ions and small molecules such as lipids [3].

The ER is a hotspot for protein folding and membrane biogenesis [4,5]. It is the entry point to the secretory pathway and contributes to the biogenesis of peroxisomes, lipid droplets, and autophagic membranes. All proteins entering the endomembrane system via the secretory pathway are synthesized by cytosolic ribosomes. In mammalian cells, the ER handles roughly 10,000 different proteins (about 30% of the proteome), thereby accumulating between 0.1 and 2.0 million client proteins every minute in some cell types [6]. Most proteins entering the secretory pathway in *Saccharomyces cerevisiae* (*S. cerevisiae*) are membrane proteins. They are co- or post-translationally inserted into the lipid bilayer and folded with the help of molecular chaperones. Probably the most prominent chaperone in the lumen of the ER is the immunoglobulin heavy-chain binding protein (BiP; GRP78; Kar2p in *S. cerevisiae*) [7]. In a similar way to all HSP70 chaperones, it binds and hydrolyzes ATP in its nucleotide binding domain (NBD), whilst undergoing cycles of binding and releasing client proteins at its substrate binding domain (SBD). This dynamic interaction with unfolded proteins prevents their unfavorable interactions and co-aggregation with other folding intermediates. Nucleotide exchange factors and J-domain-containing co-chaperones (ERdj1-8) modulate the activity of BiP and help recruit clients [7,8]. When a protein fails to fold in the ER, it can be subjected to the ER-associated degradation (ERAD) machinery and degraded via the ubiquitin-proteasome system [9]. Successfully folded proteins and properly glycosylated proteins, however, can exit the ER via COPII vesicles for their transport to the Golgi apparatus [10,11].

A carefully orchestrated machine for endomembrane trafficking guarantees the distribution of soluble and membrane proteins to their final, subcellular destination. Cargo sorting relies on specific cargo receptors, but also—in the case of membrane proteins—on the physicochemical properties of the transmembrane domains [10,11,12]. The dynamic partitioning of a transmembrane protein between an emerging transport vesicle and its donor organelle provides a means to concentrate it in one or the other compartment. The hydrophobic thickness of a transmembrane domain and its membrane environment is particularly relevant for such mismatch-based sorting mechanisms [12,13]. A gradual increase in membrane stiffness along the secretory pathway facilitates a step-by-step sorting of transmembrane proteins with increasing hydrophobic thicknesses at each station along the secretory pathway from the ER to the plasma membrane [10,12,13,14,15]. In this context, the ER membrane fulfils a special role, because it has to insert, fold and assemble all sorts of transmembrane proteins, irrespective of their largely distinct transmembrane domains (on average ~20.3/20.6 hydrophobic residues in a transmembrane helix for proteins of the early secretory compared with ~24.4/27 for proteins of the late secretory pathway in mammals/fungi) [13]. To provide a suitable environment for this diverse set of membrane proteins, the ER membrane must be particularly soft and deformable; to this end, sensitive surveillance systems keep the sterol concentration in the ER low (~5–10 mol%) [16,17] and the proportion of poorly packing, mono-unsaturated fatty acyl chains high (>70 mol%) [18].

The ER is also a hotspot of lipid biosynthesis and hosts a vast repertoire of lipid metabolic enzymes [19,20]. Lipogenic enzymes in the ER include fatty acid desaturases and elongases, as well as dozens of enzymes for producing glycerophospholipids, sterols, ceramides (the precursors for more complex sphingolipids), and neutral storage lipids [19,20]. In contrast to secretory and membrane proteins, which commute between organelles via vesicular carriers, lipids are distributed also by lipid transfer proteins [3]. However, despite the continuous and rapid exchange of membrane material via vesicular and non-vesicular transport mechanisms, each organelle of the endomembrane system pathway maintains its own, characteristic membrane properties as a means to establish its identity [21,22].

Because the ER is both structurally and functionally interconnected with essentially every cellular compartment, any disruption of ER function must have a broad impact on cellular function. It has become clear that the unfolded protein response (UPR), which is best known as a stress response to accumulating unfolded proteins in the lumen of the ER, can integrate various physiological signals to trigger an adaptive response and reestablish ER homeostasis [23,24,25,26]. Here, we review our current understanding of the early events that lead to UPR activation, with a particular focus on the role of the ER membrane and its composition. We discuss why maintaining the ER membrane stiffness via the UPR is crucial in maintaining a functional secretory pathway.

## 2. The Conventional Role of the Unfolded Protein Response (UPR) Is Proteostasis

Cells need to adapt when the folding machinery of the ER is overwhelmed and when unfolded or misfolded proteins accumulate—a situation referred to as ER stress. The UPR was originally identified as a signaling pathway that senses ER stress to upregulate ER-resident chaperones [27,28,29]. Soon, it became clear that the UPR regulates not only the folding machinery in the ER, but also the processes with relevance for the entire secretory pathway, including lipid metabolism, protein translocation, ER-associated protein degradation (ERAD), ER-to-Golgi transport and Golgi-to-ER retrieval, protein glycosylation in the ER and the Golgi apparatus, vacuolar targeting, distal secretion, and cell wall biogenesis [30]. In mammals, the UPR relies on three single-pass, transmembrane proteins in the ER; namely, the activating transcription factor 6 (present as the isoforms ATF6α/β in mammals), the inositol-requiring enzyme 1 (with two mammalian isoforms IRE1α/β), and the double-stranded RNA-activated protein kinase (PKR)—such as ER kinase (PERK) [24]. IRE1 constitutes the most conserved branch of the UPR and represents the only UPR transducer in *S*. *cerevisiae* (ScIRE1).

When activated, the UPR (1) lowers the global rate of protein production, (2) upregulates the rate of membrane lipid biosynthesis, (3) induces the production of ER-luminal chaperones and components of the ERAD machinery, and (4) expands the capacity of the secretory pathway [24]. If these adaptive responses are insufficient to restore ER homeostasis, the prolonged activity of the UPR can lead to cell death [24]. Given the broad transcriptional and non-transcriptional effector functions of the UPR, its crucial role in cell fate decisions between life, death, and differentiation, is unsurprising [24,31,32,33].

The upregulation of membrane lipid biosynthesis via UPR signals in *S. cerevisiae* causes an expansion of the ER membrane network [34,35]. Likewise, all three branches of the mammalian UPR regulate key steps of lipid metabolism and contribute to the ER membrane expansion via transcriptional and non-transcriptional mechanisms [36,37,38,39]. How the composition and properties of the ER membrane contribute to UPR activation in return has been lagging. Meanwhile, it is clear that a variety of signals originating from the ER membrane serve as potent signals for UPR activation in lipid metabolic adaptation and disease [31,40,41]. It is true, for example, that insulin-producing β-cells rely on UPR signals for their normal differentiation into professional, secretory cells [42], but it is the chronic stress caused by the excess of saturated fatty acids that kills them [43]. Before going into a more detailed discussion of the signals that lead to UPR activation, we will introduce the three branches of the mammalian UPR.

## 3. Three Musketeers—The Mammalian UPR Transducers

**Figure 1:**
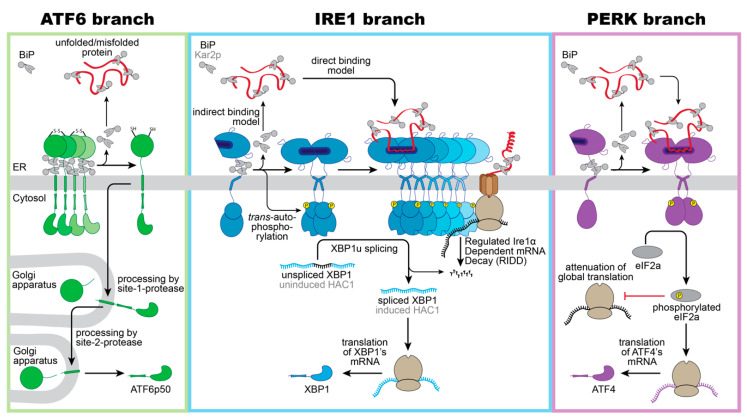
Schematic overview of proteotoxic ER stress signaling by the UPR. Three branches of the UPR sense proteotoxic stress in the ER to control adaptive transcriptional and non-transcriptional responses: ATF6, IRE1 (IRE1α in mammals/ ScIRE1 in *S. cerevisiae*) and PERK. (**Left panel**): Intermolecular disulfide bonds stabilize inactive homo-oligomers of ATF6 thereby limiting the pool of ATF6 that can be activated. In the absence of ER stress, ATF6 interacts with its negative regulator BiP. Notably, various regions of the ER-luminal domain of ATF6 contribute to BiP binding, but only a membrane-proximal binding region is indicated here for simplicity. Current models suggest a dissociation of BiP from ATF6 upon ER stress. The unmasking of Golgi localization signals allows for packaging of ATF6 into COPII vesicles for a transport to the Golgi apparatus. Processing by the site-1-protease and site-2-protease releases a transcriptionally active fragment (ATF6p50) for regulating UPR target genes in the nucleus. (**Middle panel**): Inactive monomers of IRE1α/ScIre1 associate with BiP/Kar2p via various interaction sites. Proteotoxic ER stress causes a dimerization of IRE1α/ScIre1, the release of BiP/Kar2p, and the formation of higher oligomeric assemblies of IRE1α/ScIre1. The enforced proximity of the cytosolic effector domains enables a *trans*-autophosphorylation of the cytosolic kinase domain and activation of the RNase domain. Oligomers of IRE1α and ScIre1 cleave the mRNA of unspliced XBP1 and uninduced *HAC1*, respectively, as the committed step for unconventional splicing. Translation of the spliced mRNA yields an active transcription factor XBP1/HAC1 upregulating hundreds of UPR target genes in the nucleus. Oligomers of the mammalian IRE1α oligomers can reduce the load of the ER with unfolded proteins via the regulated IRE1α-dependent mRNA Decay (RIDD). (**Right panel**): Proteotoxic ER stress causes a dissociation of BiP from PERK and facilitates the formation of PERK dimers and oligomers. *Trans*-autophosphorylation activates PERK’s cytosolic kinase domain, which then phosphorylates the eukaryotic initiation 2α (eIF2α). This causes a global attenuation of translation, but also selectively promotes the production of the transcription factor ATF4. ATF4 controls both pro-survival and pro-apoptotic signals.

ATF6 is a single-pass, type II transmembrane protein. Its C-terminal, ER-luminal domain forms intermolecular disulfide bonds that stabilize homo-oligomeric, inactive assemblies [44,45]. This way, ATF6 is directly sensitive to reducing conditions that would interfere with the normal oxidative folding in the ER. Furthermore, ATF6 is associated with the molecular ER chaperone BiP [46]. Under conditions of ER stress, BiP is released from ATF6 and unmasks two conserved Golgi localization signals [46]. BiP dissociation can be induced in vitro by supplementing ATP-Mg^2+^ to immuno-isolated complexes, yet additional factors such as co-chaperones are proposed to control ATF6 activation in vivo by regulating the ATPase cycle of BiP [47]. Following BiP dissocation, ATF6 is packaged in COPII vesicles [48] and transported to the Golgi apparatus, where it is proteolytically processed and activated via the site-1-protease and site-2-protease [49,50]. Released from its membrane anchor, the transcriptionally active ATF6p50 fragment enters the nucleus and triggers a broad transcriptional program to reestablish the protein folding homeostasis in the ER (Figure 1, left panel) [51,52,53].

A recent study suggested that the ER-resident oxidoreductase ERp18 associates with ATF6 and forms mixed disulfides specifically under conditions of ER stress to ensure optimal processing [54]. In fact, ERp18-depletion accelerates the rate ATF6 ER-to-Golgi trafficking, but causes aberrant processing and releases a non-productive fragment, which is not further processed by the site-2-protease [54].

Precisely how unfolded proteins and BiP ‘monomerize’ ATF6, despite a fully developed basic leucine zipper on the cytosolic side of the ER membrane, remains unexplored. Unlike the other two branches of the UPR, ATF6 cannot lower the flux of unfolded proteins into the ER. Instead, ATF6p50 induces an expansion of the ER membrane [37,38] and upregulates numerous genes encoding for ER chaperones, ER-luminal disulfide oxidoreductases, and ERAD components [38,51,52,53]. Notably, ATF6p50 and the transcription factor X-box-binding protein 1 (XBP1s), generated by the IRE1 branch of the UPR, act synergistically and can hetero-dimerize [51].

ScIRE1/IRE1α is a type I transmembrane protein conserved from yeast to humans. When unfolded proteins accumulate in the ER, ScIRE1/IRE1α oligomerizes [55], thereby juxtaposing the cytosolic kinase/RNase domains. This triggers the *trans*-autophosphorylation of the kinase domain and the activation of the RNase domain [28,56,57,58,59,60]. IRE1α excises a small intron from the *XBP1* mRNA and initiates an unconventional splicing reaction, which ultimately provides a template for the transcription factor XBP1s (‘s’ stands for spliced) [59,61]. After the cleavage and ejection of the intron, the two exons of the *XBP1* mRNA zipper up, form an extended stem, and become ligated by the catalytic subunit of the tRNA ligase complex RTBC [62,63]. Similarly, ScIRE1 initiates the unconventional splicing of the *HAC1* mRNA for generating an active transcription factor Hac1p (Figure 1, middle panel) [57,64]. XBP1s in mammals and Hac1p in *S. cerevisiae* control large transcriptional programs with hundreds of target genes involved in various aspects of membrane biogenesis, protein folding, trafficking, and degradation [30,52,53,65]. IRE1α can also lower the influx of proteins by degrading mRNAs associated with the ribosome-translocon complex in a process known as IRE1-dependent mRNA decay (RIDD) [66,67]. Furthermore, the downregulation of the biogenesis of lysosome-related organelles 1 subunit 1 (*Blos1*) mRNA via RIDD causes an impressive clustering of lysosomes to the perinuclear region in stressed cells, which is crucial to efficiently remove protein aggregates via late endosome-mediated microautophagy [68].

PERK is an ER-resident, type I transmembrane kinase [69]. When unfolded proteins accumulate in the ER, PERK oligomerizes and its cytosolic effector domains are activated through *trans*-autophosphorylation [69]. The activated PERK phosphorylates the eukaryotic translation initiation factor 2α (eIF2α) at serine 51, thereby rapidly inhibiting the global rate of mRNA translation and lowering the flux of proteins into the ER [69,70]. The activating transcription factor 4 (ATF4) escapes this inhibition and is selectively upregulated (Figure 1, right panel) [71]. ATF4 upregulates genes involved in amino acid metabolism, tRNA charging, and glutathione biosynthesis [72], the production of the pro-apoptotic transcription factor C/EBPP homologous protein (CHOP)—also known as growth arrest—the DNA damage-inducible gene 153 (GADD153), and the growth arrest and DNA damage-inducible gene 34 (GADD34) [71,73]. CHOP/GADD153 and GADD34 orchestrate the PERK-dependent signaling output, which can be either cytoprotective or pro-apoptotic. The cytoprotective GADD34 provides a negative feedback loop that terminates the PERK-signaling downstream of eIF2α phosphorylation, by forming a complex with the protein phosphatase 1 (PP1c) [73]. CHOP/GADD153, on the other hand, provides pro-apoptotic signals [74] by inducing the expression of the death receptor 5, leading to ligand-independent signaling via Caspase 8 [75]. The transient activation of IRE1α during acute ER-stress, on the contrary, attenuates the death receptor 5 mRNA level via RIDD, so that two opposing UPR signals control the death receptor 5 level, and thus apoptosis [75].

## 4. A Common Principle: The Oligomeric State Regulates Activity of UPR Transducers

How precisely UPR transducers can sense an accumulation of unfolded proteins in the lumen of the ER is a matter of active debate. The core principle that the dimer/oligomer formation is the basis of UPR activation, is widely accepted for IRE1α/ScIre1 and PERK [56,76,77,78,79,80,81,82,83,84]. Enforcing the homo-dimerization and homo-oligomerization of IRE1α/ScIRE1 or PERK drives UPR activation [85], whereas disrupting the interfaces for dimerization and oligomerization prevents it [86,87,88,89]. These findings established a clear link between the oligomeric state and the activity of the UPR [84,86,87]. It is expected that IRE1α/ScIRE1 and PERK use similar mechanisms of sensing, because their ER-luminal domains are structurally similar [80,86,90] and functionally equivalent [84]. The ER luminal domain of ATF6, however, is structurally unrelated and most models suggest that ATF6 needs to monomerize for its activation [44,45,91]. In the following, we focus our discussion on how IRE1α/ScIRE1 senses an accumulation of unfolded proteins in the ER.

## 5. Three Mechanisms of Sensing Unfolded Proteins by IRE1

**Figure 2:**
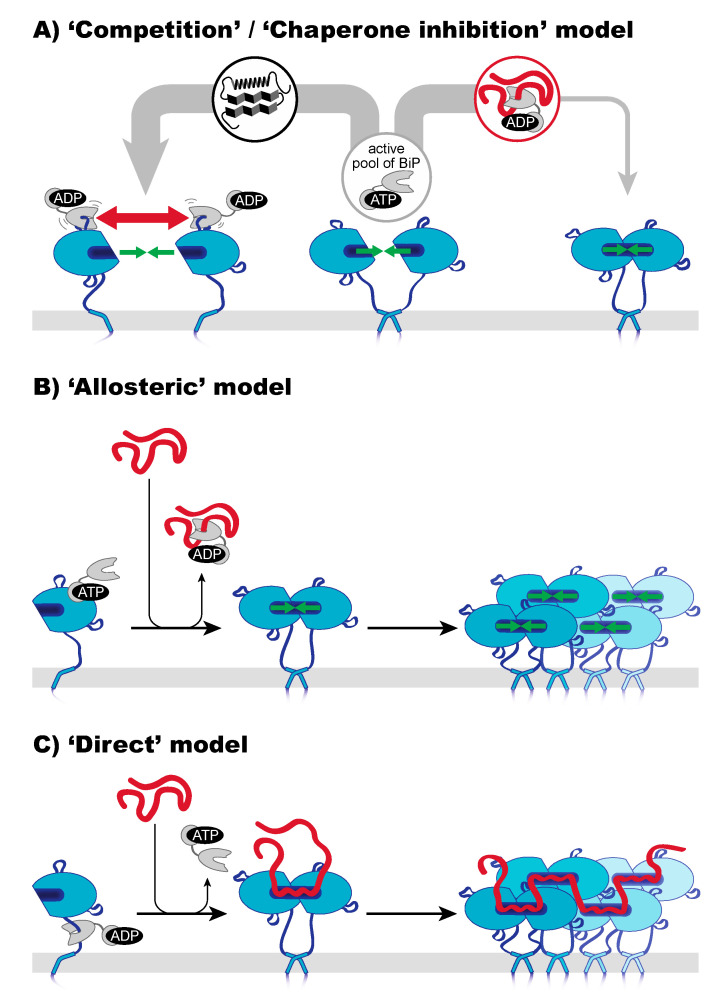
Three models of how IRE1α might sense ER stress. Currently, three models of IRE1α activation by unfolded proteins are being discussed. (**A**) The ‘competition’ or ‘chaperone inhibition’ model attributes a more passive role to the UPR transducer IRE1α and suggests that BiP acts as the true sensor of ER stress. The chaperone BiP associates dynamically with IRE1α molecules in the absence of stress and counteracts the intrinsic tendency of IRE1α to dimerize/oligomerize. Upon ER stress BiP associates with unfolded/misfolded proteins, thereby dissociating from IRE1α and enabling its dimerization and activation. (**B**) The ‘allosteric’ model suggests that BiP’s nucleotide binding domain (NBD) associates with IRE1α to sterically block its dimerization/oligomerization. The accumulation of unfolded/misfolded proteins in the lumen of the ER is sensed by BiP’s substrate binding domain (SBD). The binding of unfolded proteins to the open SBD induces a conformational change in BiP and the dissociation from IRE1α. (**C**) The ‘direct’ model suggests a direct binding of unfolded/misfolded proteins to the ER-luminal domain of IRE1α. Unfolded proteins are thought to bind to peptide-binding pockets across the dimer interface of IRE1α’s ER-luminal domain. Unfolded proteins stabilize dimeric and oligomeric assemblies of IRE1α, and therefore serve as activating ligands triggering the UPR.

Three mechanisms of how IRE1α senses ER stress have been proposed; they are not mutually exclusive and may, in fact, cooperate [92]. (i) The ‘competition’ model, recently refined as ‘chaperone inhibition’ model, proposes that the ER-luminal chaperone BiP acts as the true sensor of ER-stress and that IRE1α and unfolded proteins compete for the binding of BiP. According to this model, BiP is required to maintain IRE1α in a monomeric, inactive state. When unfolded proteins accumulate, however, BiP is titrated away from IRE1α, thereby unleashing IRE1α’s inherent ability to dimerize for UPR activation (Figure 2A) [93,94,95]. (ii) The ‘allosteric’ model suggests that BiP uses its NBD to block the oligomerization of IRE1α [92,96]. According to this model, it is the binding of an unfolded protein to BiP which triggers the dissociation of the BiP-IRE1α complex as a prerequisite for IRE1α activation (Figure 2B) [97]. (iii) The ‘direct’ model suggests that unfolded proteins interact directly with ScIRE1 [86,98] and IRE1α [89] thereby stabilizing dimeric and higher oligomeric assemblies of IRE1α/ScIRE1 that provide a platform for UPR signaling (Figure 2C) [99]. Although the ‘indirect’ and ‘direct’ models make clear predictions on the nature of sensing, it remains challenging to assess their relevance in vivo [97,99,100].

## 6. A Closer Look on Indirect Models of Sensing—Co-Opting BiP as a Sensor

Indirect models predict a causal, inverse correlation between BiP association and the oligomeric state of IRE1α. Historically, this was based on the observations that less BiP is co-immunoprecipitated with IRE1α from stressed cells and that an enforced production of BiP renders cells more resistant to ER stress, whilst lowering UPR signaling in response to proteotoxic challenges [84,101]. The finding that not only IRE1α, but also PERK and ATF6 are co-immunoprecipitated with BiP supported the view that BiP may act as a general sensor helping UPR transducers to sense unfolded proteins [46].

The model of a highly dynamic, ‘chaperone inhibition’ model was fueled by the recent finding that BiP is recruited to IRE1α by the J-domain-containing protein ERdj4 [93,94]. Analogous to canonical chaperone-client interactions, BiP uses its SBD to bind IRE1α and counteracts an inherent tendency of IRE1α to dimerize [80,95]. In the ATP-bound state, BiP is recruited to IRE1α by ERdj4, which also stimulates ATP hydrolysis in BiP’s NBD [93]. The resulting ADP-bound BiP interacts more stably with IRE1α thereby locking it in a monomeric state [93,94]. An intriguing aspect of this model is that BiP’s ability to counteract IRE1α dimerization serves as a proxy for its ability to maintain protein folding homeostasis in the ER [100]. The sensitivity of the UPR can therefore be adjusted even if the influx of unfolded proteins into the ER is unchanged. A decrease in the ER-luminal Ca^2+^ concentration, for example, favors the formation of inactive BiP oligomers, which would lead to UPR activation even without a need for an accumulation of unfolded proteins [102]. Notably, a specific functional role for higher oligomeric assemblies of IRE1α has not been discussed in the framework of this model [94,100,102].

If BiP is indeed the principal regulator of UPR activation, then the removal of BiP binding sites from IRE1α should lead to an uncontrolled, chronic activation of the UPR. An engineered, ‘BiP-less’ variant of IRE1α that barely co-immunoprecipitates with BiP, however, is still responsive to ER stress-inducing agents and causes prolonged durations of UPR signaling [103]. Analogous experiments and observations have also been made in *S. cerevisiae* [104,105]. Although these findings suggest that BiP may not be the sole, dominant regulator of UPR activity, it is also clear that co-immunoprecipitation experiments are ‘blind’ for dynamic, transient interactions. In fact, recent data suggest that such transient interactions between BiP and flexible loops of IRE1α’s ER-luminal domain contribute to the regulation of IRE1α [94].

The ‘allosteric’ model suggests, based on in vitro data, that BiP interacts with IRE1α via its NBD to prevent a dimerization of IRE1α. Central to this model is that the binding of unfolded proteins to the SBD of BiP triggers the dissociation of the BiP-IRE1α complex. A potential binding region for BiP’s NBD on IRE1α was identified by hydrogen-deuterium exchange experiments [94] and awaits further in vivo characterization. Recent in vitro data show that BiP can modulate the oligomeric state of IRE1α even in the absence of unfolded proteins [94], thereby rendering a key aspect of this model unnecessary; however, this does neither exclude a direct interaction of BiP’s NBD with IRE1α nor the regulatory potential of this mechanism. IRE1α co-immunoprecipitates also with the J-domain-containing protein Sec63/ERdj2 of the ER protein translocation machinery where BiP acts as a ratchet for protein transport [106], and therefore it will be fascinating to learn how the Sec63/Erdj2-dependent activation of BiP contributes to the regulation of IRE1α by resembling the mechanism of other HSP70-type chaperones in protein translocation [107].

## 7. A Closer Look on the Direct Model—Unfolded Proteins as Agonists

The first evidence for a ‘direct’ sensing of unfolded proteins by an UPR transducer came from the structural work on the conserved core region of ScIRE1 [86]. A deep hydrophobic pocket resembling the peptide binding groove of the major histocompatibility complex (MHC) [108], extending across the interface between two neighboring protomers, suggested that unfolded proteins may act as direct, activating ligands for ScIRE1 by stabilizing dimeric and/or higher oligomeric assemblies [86]. In fact, the isolated, core ER-luminal domain of ScIRE1 possesses anti-aggregation activity in vitro and interacts with the peptides derived from misfolded proteins with micromolar affinities [98,109]. The binding of such peptides also promotes the formation of higher oligomers of the core conserved region in vitro, and therefore it is also likely that unfolded proteins exposing one or more hydrophobic patches stabilize higher oligomeric assemblies of ScIRE1 in vivo. Additional observations disfavored a dominant, indirect mode of sensing via Kar2p. The removal of the major Kar2p binding site from ScIRE1 in the juxtamembrane, intrinsically disordered the region results in a construct with a low, basal activity, which remains responsive to ER stress [104,105]. Moreover, even though additional, short-lived interactions of Kar2p with other regions of ScIRE1 cannot be formally excluded, the identification of an autoinhibitory region in the non-conserved N-terminal portion of ScIRE1 [110] and in vivo FRET data [104] suggest that Kar2p is not the sole, master regulator of the UPR [40,99,111] and that unfolded proteins act as agonists of ScIRE1 for UPR activation.

Additional crystal structures of the ER-luminal domains of human IRE1α [80] and PERK [90] revealed a similar, overall architecture as observed in ScIRE1 including a hydrophobic groove across the dimer interface. However, the groove in the human IRE1α appeared too narrow to accommodate an unfolded polypeptide chain [80]. In line with this finding, the isolated core ER-luminal domain of human IRE1α does not possess the same in vitro anti-aggregation activity as observed for ScRE1 [87,109]. More recent biochemical and structural work on IRE1α via nuclear magnetic resonance (NMR) revealed a structural flexibility in and around the putative binding groove [89]. This suggested that the crystallized form of IRE1α may represent a ‘closed’ state, which can switch to an ‘open’ state for the binding of unfolded proteins. Using peptide-tiling arrays, it was possible to identify peptides that interact with IRE1α’s core luminal domain with a low micromolar affinity [89]. The binding of the peptide induced a conformational change in IRE1α that licenses the oligomerization of the IRE1α’s core luminal domain [89]. The dynamics and structural arrangement of full-length IRE1α in cells was also studied via confocal microscopy [55] and by super-resolution microscopy combined with single particle tracking, and photoconversion [78]. These studies demonstrate that the formation of signaling-active clusters is conserved from yeast and humans. Despite the current discussions regarding the precise binding site for unfolded proteins either inside [89] or outside the MHC-like binding groove [90], it seems clear that the core ER-luminal domain of human IRE1α can interact directly with unfolded proteins [77,89,94]. Whereas IRE1α’s affinity for such peptides is comparable to the range of affinities reported between molecular chaperones and their clients [112,113], it is notable that the peptide binding preference of human IRE1α and BiP are distinct [99]. This means that IRE1α and BiP do not compete for the same set of peptides. These differences in the binding preference may provide a handle to dissect the relative contributions of ‘direct’ and ‘indirect’ sensing mechanisms to UPR activation in the future.

Despite significant advances, it remains exceedingly challenging to recapitulate the molecular events that lead to UPR in vitro. UPR transducers maintain complex, dynamic interactions with numerous regulatory proteins on both sides of the ER membrane (referred to as UPRosomes) [23,114]. Not all ER-luminal chaperones, for example, are negative regulators of the UPR. The chaperone HSP47, known for its role as a collagen-specific chaperone [115], acts as a positive modulator of the UPR, by stripping BiP away from IRE1α [116]. Analogously, it has been discussed that some unfolded proteins may even expose regions that counteract the oligomerization of IRE1 or PERK by blocking either dimerization or oligomerization interfaces [83]. The protein disulfide isomerase A6 (PDIA6), on the other hand, regulates the duration and strength of UPR signaling [117]. It attenuates UPR activity and counteracts stress-induced apoptosis by directly binding to a cysteine in IRE1α’s ER-luminal domain, which is normally oxidized upon activation [117]. Notably, even the cytosolic kinase domain of ScIRE1 seems to bear important regulatory functions by sensing the cytosolic ADP level as a proxy for the energy status of the cell [40].

A picture emerges in which IRE1α/ScIRE1 forms complex interactions with the entire machinery involved in the production, translocation, and folding of proteins at the entry point of the secretory pathway [67]. The intricate connection between IRE1α, the ribosome [67,118], and the Sec61/Sec63 translocon [77,103,119], together with IRE1α’s ability to degrade mRNAs via RIDD [66], provides all ingredients for a selective degradation of mRNAs encoding for those proteins, which are particularly problematic to fold such as multidomain membrane proteins. The recent observation from in situ cryo-electron microscopy, that signaling-active clusters of IRE1α remodel the ER membrane with the MHC-like binding groove pointing towards the surface of the ER membrane, puts a spotlight on the role of the ER membrane in controlling UPR activity [120].

## 8. A Conserved Sensitivity of UPR Transducers for Lipid Bilayer Stress

The UPR counteracts the accumulation of unfolded proteins in the ER lumen [27], but also links numerous lipid metabolic ER functions to insulin signaling and glucose metabolism [121]. It is stunning that such a powerful response with hundreds of target genes fails to overcome metabolic challenges, as observed in the context of obesity, metabolic syndrome, and aberrant lipid management [122]. Chronic ER stress develops when cells fail to adapt to the continuous presence of the stress-inducing agent/metabolite, or when the UPR signaling aggravates the stress that was caused by the original metabolic insult [31,122,123]. An oversupply of saturated fatty acids, for example, causes lipotoxicity [124,125], which is also associated with changes in the ER membrane composition and structure [125]. Although metabolic and transcriptomic analyses identified the UPR as a key target of lipotoxicity [124], the contribution of a membrane-based UPR to health and disease remains understudied. Complex metabolic diseases associated with chronic ER stress, such as diabetes [126,127] and non-alcoholic fatty liver disease [128], have characteristic, cellular lipid fingerprints [129], but their mechanistic and physical role in perpetuating UPR signaling via membrane-based signals remains challenging to study.

In recent years, it has become clear that aberrant ER membrane compositions, collectively referred to as lipid bilayer stress (LBS), can potently and directly activate the UPR. This membrane-based activation of the UPR is evolutionary conserved and has been described in yeast [130,131,132], worms [132], and mammals [133,134]. There is compelling evidence that LBS acts directly on UPR transducers without a need for activating signals from unfolded proteins in the ER lumen. UPR transducers remain responsive to LBS even when the entire core sensory ER-luminal domain is removed [134,135]. It was demonstrated that aberrant stiffening of the ER membrane stabilizes dimers and potentially also oligomers of ScIre1 thereby causing UPR activation [136,137]. However, under most conditions of LBS, less oligomerization of IRE1α/ScIRE1 is observed compared with the conditions of proteotoxic stress despite a similar degree of *XBP1*/*HAC1* mRNA splicing [133,135,136,138]. The evolutionary conserved, dual sensitivity of UPR transducers to proteotoxic stress and LBS suggests a broader role of the UPR beyond the homeostasis of protein folding in the ER. Deciphering the relative contribution of unfolded proteins and LBS to UPR activation will be important. The use of 4-phenylbutrate (4-BPA), which is often referred to as a chemical chaperone [131,139,140,141], can be misleading in this context, despite its potency to restore glucose homeostasis in a mouse model of type 2 [142]. The recent finding that 4-BPA attenuates ER retention by directly binding to Sec24 of the COPII machinery suggests that it does not act directly on unfolded proteins, but instead on membrane traffic [143]. We are convinced that the UPR acts as a guardian of the secretory pathway that surveys all secretory and membrane material entering the ER.

## 9. A Stunning Variety of Signals Cause Lipid Bilayer Stress

**Figure 3:**
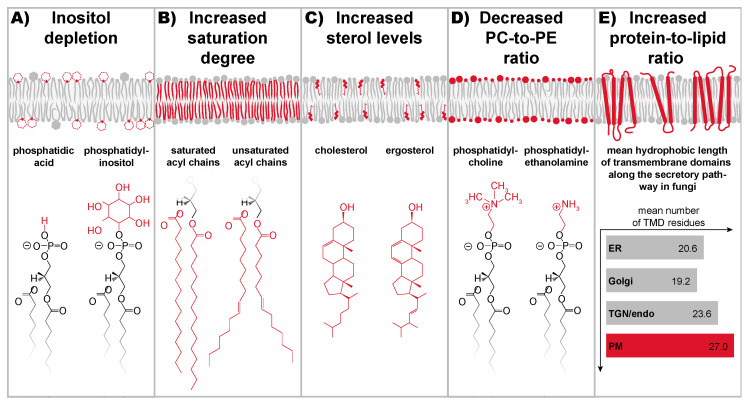
Overview of conditions causing lipid bilayer stress (LBS). Various perturbations of lipid metabolism cause LBS and provide potent signals for UPR activation. (**A**) The depletion of inositol reduces the level of phosphatidylinositol (PI) lipids and causes a robust, but transient activation of the UPR via a membrane-based mechanism. (**B**) An increased level of tightly packing lipids with two saturated fatty acyl chains stiffens the ER membrane and activates the UPR. (**C**) Increased sterol levels in the ER cause ER membrane stiffening and UPR activation. (**D**) A decreased PC-to-PE ratio directly activates the UPR by an unknown mechanism. An increased PC-to-PE level has also been associated with chronic ER stress, but seems to act indirectly via an impact on protein folding. (**E**) The accumulation of plasma membrane proteins with thick, hydrophobic transmembrane domains in the ER, causes a stretching of the fatty acyl chains in ER membrane lipids. We have proposed that an overcrowding of the ER with membrane proteins of the late secretory pathway, exhibiting a higher average number of hydrophobic transmembrane residues, causes LBS and UPR activation [136].

Systematic genetic screens in *S. cerevisiae* revealed an intricate crosstalk between the lipid metabolic network and the machinery involved in protein folding, degradation, and trafficking [135,144,145]. Even though relatively few conditions of LBS have been investigated in mechanistic detail, it is becoming increasingly clear that a variety of lipid metabolic perturbations, targeting structurally and chemically distinct components of the ER membrane, cause LBS (Figure 3).

Inositol was the first lipid metabolite implicated in UPR activation [146,147]. As an abundant lipid building block, inositol is found in various lipids including phosphatidylinositol (PI) (Figure 3), phosphatidyl-inositol-phosphates, and yeast-specific sphingolipids [19,148]. Inositol-depletion causes a robust, but transient activation of scIRE1 [137,146,149,150]. This suggests that the UPR remodels the lipid metabolic network sufficiently to counteract LBS and to reestablish ER membrane homeostasis. Although inositol-depletion is only used routinely in *S. cerevisiae*, the great potential of dietary inositol in the context of human diseases associated with chronic ER stress has been recently highlighted in an excellent review [151]. In *S. cerevisiae*, inositol-depletion activates ScIRE1 directly via membrane-based signals, and apparently without causing significant protein misfolding in the ER [136,138,146,149,150]. The diffusion of the ER-luminal chaperone Kar2p is slowed down upon proteotoxic stress due to interactions with its unfolded/misfolded clients, but it remains unaffected by inositol-depletion [149]. The point mutations that render ScIRE1 virtually insensitive to LBS whilst conserving its ability to respond to proteotoxic stress provides a means to distinguish their relative contribution to UPR activation [135,136,138,150,152]. However, the precision with which inositol-depletion affects the molecular composition of the ER membrane and its physicochemical properties, remains to be elucidated.

Saturated fatty acids cause lipotoxicity [125]. A variety of mechanisms have been proposed by which saturated fatty acids may cause cellular stress [121], including the production of ceramides [153], reactive oxygen species [154], and diacylglycerols [155]. Particularly important is the LBS caused by membrane lipids with saturated fatty acyl chains [125,133,134,145,156,157] (Figure 3). This view is supported by systematic transcriptomic and metabolic analyses that identified the UPR as a major target of lipotoxicity [124]. Increased levels of membrane lipid saturation stiffens the ER membrane, disrupts its structure [125,158], and activates the UPR [131,145]. Notably, IRE1α and PERK remain sensitive to the stress caused by palmitate even when the entire ER-luminal domain is removed and when the transmembrane helix is exchanged by the transmembrane helix of an unrelated protein [134,159]. Significantly, this membrane-based activation of the UPR might trigger a vicious cycle that perpetuates the disparity between saturated and unsaturated lipids [41]. However, various unsaturated fatty acids counteract lipotoxicity by increasing the flux of fatty acids into storage lipids [160,161], and by establishing a new balance between saturated and unsaturated lipids in cellular membranes [145,162].

One of the most important factors contributing to membrane stiffness and lipid packing in eukaryotic cells are sterols (Figure 3). Normally, the sterol level of the membrane ER is kept low [17] by a collection of remarkably sensitive mechanisms [16,163,164]. However, aberrantly increased sterol levels in the ER cause UPR activation in both yeast and mammals [131,165,166]. Abundant, free cholesterol from advanced atherosclerotic lesions, for example, is taken up by macrophages and stiffens the ER membrane, causing chronic ER stress that can ultimately lead to apoptosis [166]. Strikingly, the inhibition of IRE1α counteracts the progression of atherosclerosis [167]. Although structurally distinct, sterols and saturated fatty acids act synergistically in UPR activation as shown in vivo [131] and in vitro [136]. As a collective, they determine ER membrane stiffness, which ultimately controls the oligomeric state of UPR transducers.

Phosphatidylcholine (PC) and phosphatidylethanolamine (PE) are among the most abundant glycerophospholipids in *S. cerevisiae* and mammals, and are therefore important determinants of the collective physicochemical properties in cellular membranes [10,20,168]. PC has a larger hydrophilic head group than PE; perturbations of the PC-to-PE ratio change the lateral pressure profile of the membrane and affect the structural dynamics and function of virtually every integral membrane protein [169,170,171,172] (Figure 3). Severe, chronic ER stress and massive UPR activation is observed when the PC-to-PE ratio is decreased by deleting the gene for a methyl-transferase required to generate PC lipids from PE as demonstrated in *S. cerevisiae* and mammals by an *OPI3* and the PEMT^−/−^ knockout, respectively [173,174]. Notably, ScIRE1 is activated in *OPI3* knockout cells even when its entire ER-luminal domain is removed [135], thereby suggesting that signals from the ER membrane, potentially ER membrane stiffening, trigger the UPR directly. A low PC-to-PE ratio has ripple effects throughout the lipid metabolic networks and also causes an increase in lipid saturation and a decrease in membrane fluidity [175,176,177]. It is unlikely, however, that changes in membrane fluidity act as UPR activating signal; a decreased fluidity of the ER membrane would slow down both the association and the dissociation of UPR transducers without an impact on the equilibrium constant for homo-oligomerization. Intriguingly, choline supplementation counteracts the severe ER stress observed in *OPI3* knockout cells [135,173,177]. Likewise, a choline-enriched diet reverses the liver damage of PEMT^−/−^ mice [178]; even an increased PC-to-PE ratio as observed in obese mice causes chronic UPR activation and steatohepatitis [179]. In this case, it was suggested that the abnormally high PC-to-PE ratio impairs the ER-localized Ca^2+^ pump SERCA, thereby lowering the ER-luminal Ca^2+^ level and impairing the function of Ca^2+^-dependent chaperones including BiP [102,179]. It appears that an aberrant PC-to-PE ratio, either increased or decreased beyond a certain range, promotes ER stress. Nevertheless, only a reduced PC-to-PE ratio represents a condition of LBS, because it directly activates the UPR via a membrane-based mechanism [135,174].

Sphingolipids form a large group of ceramide-containing lipids. Ceramides are synthesized in the ER and further modified in the Golgi apparatus to form complex sphingolipids [180]. Increasing sphingolipid production in *S. cerevisiae* by deregulating the rate-limiting step of sphingolipid biosynthesis leads to a chronic activation of the UPR. The respective cells exhibit an increased cellular sensitivity to ER stress and a hampered ER-to-Golgi transport [181,182,183]. Likewise, compromising the utilization of very long chain fatty acids (VLCFAs) by a *FAT1* deletion sensitizes cells to ER stress, increases the level of the sphingolipid metabolic intermediate phytosphingosine, and causes somewhat elevated levels of saturated membrane lipids [184]. However, it remains hard to pinpoint how exactly the *FAT1* knockout activates ScIRE1, because VLCFAs are required not only for the production of sphingolipids, but also for GPI anchors, phosphatidylinositol, and storage lipids [185]. Even though the molecular mechanisms by which sphingolipids modulate UPR activity remain to be elucidated, it is becoming increasingly clear that sphingolipid metabolism and the ER stress response are tightly intertwined in *S. cerevisiae*. The mammalian UPR transducer ATF6 has been reported to bind directly via its transmembrane helix to two intermediates of the ceramide biosynthetic pathway: dihydrosphingosine and dihydroceramide [186]. This interaction is thought to serve as an activating signal for the packaging of ATF6 in COPII vesicles [186]. The vesicular transport from the ER to the Golgi apparatus is regulated by specific sphingolipids [187], and therefore it will be interesting to further dissect the crosstalk of sphingolipid biosynthesis, ER-to-Golgi transport, and the membrane-based UPR.

Hopefully, these examples have illustrated that a large variety of lipid metabolic perturbations trigger the UPR. For some of these examples it is already clear that the signal for UPR activation comes directly from the membrane. To establish the fingerprints of a stressed ER, it will be of utmost importance to obtain quantitative information on the ER membrane composition of unstressed, stressed, stress-adapted, and chronically stressed cells. At this moment, it seems that an increased ER membrane stiffness is the common denominator of LBS [21].

## 10. How UPR Transducers Sense Membrane Stiffening from Lipid Bilayer Stress

Any major change of the ER membrane composition will affect in one way or another the collective physicochemical properties of the ER membrane, such as the bending rigidity, membrane stiffness, or membrane fluidity, which in turn affect all ER membrane proteins. This is exemplified by the Ca^2+^ pump SERCA, which is functionally compromised both by increased cholesterol levels and an increased PC-to-PE ratio in the ER [166,179]. If UPR transducers serve a dedicated role in controlling a specific physicochemical property of the ER, they should bear specific structural features that render them more sensitive to this very property than other proteins [41]. Most of our mechanistic understanding on how aberrant membrane stiffening of the ER is recognized by the UPR transducers is based on studies of ScIRE1 [135,136,137].

**Figure 4:**
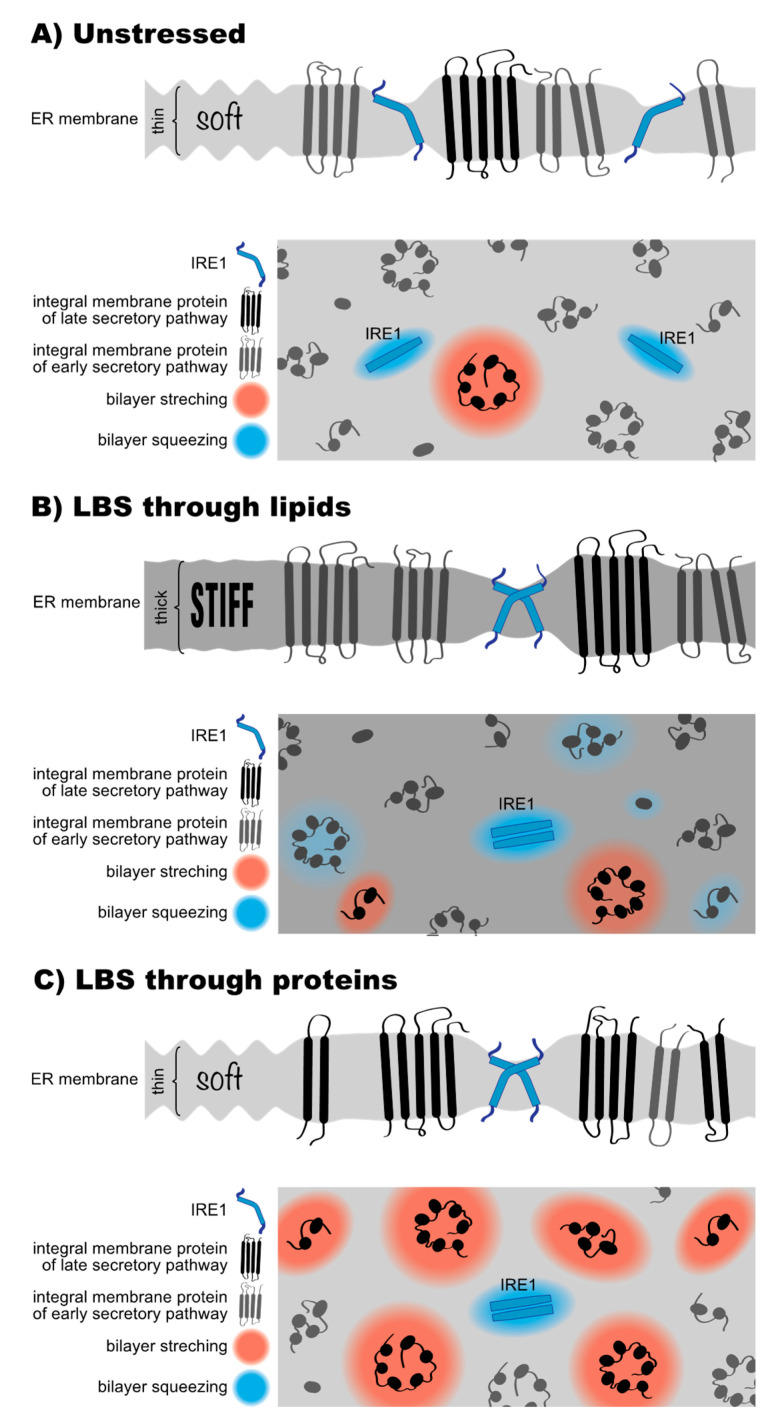
Models for lipid bilayer stress sensing by IRE1. (**A**) The lipid composition of the ER is optimized for accepting all sorts of transmembrane proteins, including plasma membrane proteins (black) with an increased hydrophobic thickness that locally stretches the lipid bilayer to overcome the hydrophobic mismatch (red shade). ScIRE1 (blue) has an unusually short transmembrane helix equipped with ER-luminal amphipathic helix. This unusual transmembrane region causes a local compression of the lipid bilayer to overcome the negative hydrophobic mismatch (blue shade). (**B**) Aberrant lipid compositions of the ER membrane can cause bilayer stiffening, which in turn increases the energetic costs for any hydrophobic mismatch. The unusual transmembrane architecture of ScIRE1 renders it particularly sensitive to ER membrane stiffening. The total area of membrane compression around ScIRE1 is minimized by a membrane-driven dimerization/oligomerization, which also activates the UPR. (**C**) Issues with protein folding in the ER or problems in the secretory pathway can cause an accumulation of proteins of the late secretory pathway (black) in the ER. The local stretching of the lipid bilayer (red shade) increases the local membrane stiffness thereby creating ‘no-go-areas’ for ScIRE1. The effective up-concentration of ScIRE1 and an increased membrane stiffness trigger the UPR by forcing ScIRE1 in dimers/oligomers.

The transmembrane region of ScIRE1 has two intriguing features; firstly, an unusually short transmembrane helix and secondly, an amphipathic helix on the ER-luminal side adjacent to the transmembrane helix [136]. Molecular dynamics simulations and electron paramagnetic resonance (EPR) spectroscopy have revealed that the amphipathic helix inserts deep in the lipid bilayer and forces ScIRE1′s transmembrane helix into a highly tilted and bent configuration [136,137]. This configuration is maintained even in signaling-active clusters of ScIRE1 [137]. Any mutation that disrupts this configuration also disrupts ScIRE1′s sensitivity to LBS in vivo [137]. This transmembrane architecture, but not the precise amino acid sequence, causes a local ‘squeezing’ of the ER membrane associated with lipid acyl chain disordering, to accommodate for the hydrophobic mismatch between ScIRE1 and the ER membrane (Figure 4A). The energetic penalty associated with this membrane deformation is negligible in the unstressed ER. This is because the ER membrane is extremely soft and optimized to accept all sort of transmembrane proteins with most distinct hydrophobic thicknesses. The energetic costs for the hydrophobic mismatch-based membrane compression become significant, however, when the stiffness of the ER membrane increases during LBS (Figure 4B). Under these conditions, ScIRE1 is driven together via a membrane-based mechanism into dimeric and potentially higher oligomeric assemblies, thereby coalescing and minimizing the unfavorable area of membrane compression around ScIRE1 [136,137] (Figure 4B). Given that key functions of the ER, including protein translocation [188], membrane protein extraction by the ERAD machinery [189], and protein sorting along the secretory pathway [10,190] are affected by an aberrant membrane stiffening, we believe it is no coincidence that the UPR is sensitive to this very property. Whenever the composition of the ER causes an aberrant stiffening and these central ER functions are at risk, the UPR upregulates lipid biosynthesis and slows down global protein synthesis, to lower the protein-to-lipid ratio and to ‘soften’ the ER membrane [41,151]. Notably, the amphipathic helix of ScIRE1 is also involved in sensing an aberrantly low PC-to-PE ratio. A R537Q mutation on the hydrophilic side of the amphipathic helix renders ScIRE1 insensitive to this form of LBS [135]. It therefore seems possible that not only an aberrant stiffening of the ER membrane, but also perturbations in the lipid headgroup region affect the activity of the UPR.

Significant efforts have been undertaken to dissect the mechanism by which the mammalian UPR transducers IRE1α and PERK sense LBS [134,159,191]. The short transmembrane helix and the adjacent amphipathic helix described for ScIRE1 are conserved in these mammalian UPR transducers, and therefore it is tempting to assume a similar mechanism of sensing; however, mutations in the ER-luminal amphipathic helix of IRE1α failed to disrupt IRE1α’s sensitivity to the LBS caused by saturated fatty acids [134]. Another study suggested that the differences in lipid saturation would be sensed by the transmembrane helix of IRE1α by stabilizing distinct rotational configurations, thereby mimicking the sensory mechanism of the lipid saturation sensor Mga2 from *S. cerevisiae* [18,191,192]. However, because the entire transmembrane helix of IRE1α and/or PERK can be scrambled or replaced without compromising the sensitivity for LBS [134], it is unlikely that specific structural features, such as aromatic residues in specific positions, contribute to the mechanism of sensing.

Hence, the mechanism by which IRE1α and PERK sense LBS remains unknown, despite a tremendous biomedical relevance. It is possible that the placement of the ER-luminal amphipathic helix is somewhat different for ScIRE1, IRE1α, and PERK thereby modulating the relative contribution of the hydrophobic and the hydrophilic face to LBS sensing. This would be reminiscent of the broad spectrum of strategies employed by other sensory amphipathic helices involved in regulating lipid metabolism [193,194,195,196,197,198]. The precise placement of the amphipathic helices relative to the lipid bilayer affects their sensitivity to lipid packing, whereas membrane binding is dominated by hydrophobic interactions for some proteins and by electrostatic interactions for others [193,194,195,196,197,198]. Another reason why mammalian UPR transducers might use a slightly different mode of interrogating the ER membrane than ScIRE1, is that PC lipids are more abundant in mammalian cells compared with *S. cerevisiae*, where PI lipids are more prominent [10,168,199]. Different membrane environments have likely provided different evolutionary constraints leading into different strategies of sensing LBS.

Dissecting the role of IRE1α’s amphipathic helix in LBS will be challenging because this region of IRE1α is also involved in an interaction with the Sec61/Sec63 translocon [103,200]. Protein translocation can also be blocked by an aberrant membrane stiffening [177,188], and therefore it will be interesting to learn if the UPR coordinates membrane protein insertion and folding with a physicochemical ER membrane homeostasis by balancing the rate of protein and lipid production.

## 11. What Is Proteotoxic Stress and What Is Lipid Bilayer Stress after All?

UPR transducers exhibit a conserved sensitivity to proteotoxic stress and LBS, but little is known about the relative contribution of these stresses to the UPR activity under normal physiological and pathophysiological conditions. Classically, the UPR has been studied using proteotoxic drugs such as dithiothreitol (DTT) or Tunicamycin (TM), interfering with disulfide bridge formation and the N-linked glycosylation, respectively. Hence, the activation of the UPR in response to DTT or TM is generally assumed to be driven by protein unfolding in the lumen of the ER. Mutational and functional studies in *S. cerevisiae* suggest that this is indeed the case, but only within the first hour of treatment [150]. The prolonged presence of proteotoxic stressors activate the UPR via a membrane-based mechanism. When ScIRE1 is de-sensitized to unfolded proteins by the so-called ΔIII mutation [87,150] or by a mutation that prevents the formation of higher oligomeric clusters [86], it can still mount a full-blown UPR after prolonged treatments with DTT or TM [136,137,150]. However, when ScIRE1 is rendered insensitive to both unfolded proteins and LBS, by combining the ΔIII mutation with a mutation disrupting the membrane-sensitive amphipathic helix, the UPR is muted and the respective cells are as hypersensitive to proteotoxic drugs as *IRE1* knockout cells [136,137]. These observations are not specific to *S. cerevisiae*, because even when the ER-luminal domains of IRE1α or PERK are substituted by a leucine zipper, they remain responsive to prolonged treatments with TM [85]. We conclude that prolonged cellular treatments with proteotoxic drugs can cause membrane-based stresses in both *S. cerevisiae* in mammalian cells. The molecular and physical basis, however, remains to be studied.

We proposed that membrane stiffening from an overcrowding of the ER with membrane proteins can activate the UPR [41]. This mechanism may act in parallel and synergistically with LBS-causing perturbations of the lipid metabolic network (Figure 3) [41]. A retention of misfolded membrane proteins in the stressed ER would lead to an increased protein-to-lipid ratio (Figure 4C), because lipids can leave the ER via lipid transfer proteins. The unusual transmembrane region of ScIRE1, however, is prone to sensing an increase in the protein-to-lipid ratio. By squeezing the membrane, ScIRE1 senses membrane stiffness [136], which is also increased when proteins of the late secretory pathway get stuck in the ER (Figure 3E and Figure 4C). These proteins have particularly long, hydrophobic transmembrane domains, which tend to stretch the ER membrane. A transmembrane helix of an ER protein has on average ~21 hydrophobic residues compared with ~27 in a plasma membrane protein (Figure 3E). By stretching the ER membrane, plasma membrane proteins stretch and stiffen the ER membrane, thereby creating ‘no-go-areas’ for ScIRE1 excluding the UPR transducer due to a drastic hydrophobic mismatch (Figure 4C). ScIRE1 would effectively be concentrated in the remaining, accessible regions of the crowded ER. Especially when combined with changes in the ER membrane lipid composition that increase membrane stiffness (Figure 4B) [134,136], this would provide a strong, membrane-based signal for UPR activation.

The potential of late secretory pathway proteins to overpopulate the ER during prolonged ER stress should not be underestimated, even if the ERAD machinery would remove some of them [9]. In rapidly growing cells such as the baker’s yeast, the entire repertoire of membrane proteins is duplicated with every cell division (~90 min). A severe proteotoxic stress lasting for 180 min and longer, for example, can be expected to substantially remodel the ER membrane proteome. In a similar scenario, UPR activation by an accumulation of late secretory pathway proteins in the ER, can be induced by mutations that interfere with vesicular traffic along the secretory pathway [144]. This is best illustrated by *ERV14* knockout, which delays the ER-to-Golgi transport of proteins with long transmembrane domains [12,144] and which also leads to a massive activation of the UPR [12,201]. Likewise, blocking the formation of COPI vesicles with Brefeldin A causes a disassembly of the Golgi apparatus [202], blocks secretion, and triggers the UPR [203]. Hence, it seems plausible that mis-localized proteins, independent of whether they are folded or misfolded, can trigger a membrane-based UPR (Figure 4C). To better understand their mode of action and how they synergize with the lipid matrix, it will be important to obtain quantitative information on both the ER lipid and protein composition from stressed and unstressed cells. A careful analysis of the role of membrane proteins and lipids on membrane stiffness might reveal a physical basis for chronic ER stress.

## 12. Concluding Remarks

Crucial cellular processes such as protein folding, the self-repairing of biological membranes, and the recognition of unfolded proteins by chaperones, are driven by collective physicochemical properties and simple principles. The UPR has a dual sensitivity for both unfolded proteins [24] and aberrant ER membrane stiffening [41,136] to control the biosynthetic rates of lipids, secretory proteins, and membrane proteins. Understanding how the UPR transducer senses an overcrowding of the ER membrane to balance the production of proteins and lipids is likely to establish new angles of attack to treat complex metabolic diseases associated with chronic ER stress, such as obesity, insulin resistance, diabetes, and non-alcoholic fatty liver disease. It will be crucial to establish the relative contribution of unfolded proteins and aberrant membrane stiffening to UPR activation *in situ*. Hence, there is a great need for biosensors and non-invasive tools, which provide information on the level of unfolded/misfolded protein in the ER. Likewise, it is crucial to obtain quantitative information on the ER membrane composition from stressed and unstressed cells. Given that proteotoxic ER stress and LBS converge in similar configurations of ScIre1′s transmembrane region [137], it will be fascinating to learn how different forms of ER stress establish different transcriptional programs [135].
